# QIMCMDA: MiRNA-Disease Association Prediction by q-Kernel Information and Matrix Completion

**DOI:** 10.3389/fgene.2020.594796

**Published:** 2020-10-22

**Authors:** Lin Wang, Yaguang Chen, Naiqian Zhang, Wei Chen, Yusen Zhang, Rui Gao

**Affiliations:** ^1^School of Mathematics and Statistics, Shandong University, Jinan, China; ^2^School of Control Science and Engineering, Shandong University, Jinan, China

**Keywords:** microRNA-disease interaction, association prediction, heterogeneous omics data, q-kernel neighborhood similarity, matrix factorization

## Abstract

Studies have shown that microRNAs (miRNAs) are closely associated with many human diseases, but we have not yet fully understand the role and potential molecular mechanisms of miRNAs in the process of disease development. However, ordinary biological experiments often require higher costs, and computational methods can be used to quickly and effectively predict the potential miRNA-disease association effect at a lower cost, and can be used as a useful reference for experimental methods. For miRNA-disease association prediction, we have proposed a new method called Matrix completion algorithm based on q-kernel information (QIMCMDA). We use fivefold cross-validation and leave-one-out cross-validation to prove the effectiveness of QIMCMDA. LOOCV shows that AUC can reach 0.9235, and its performance is significantly better than other commonly used technologies. In addition, we applied QIMCMDA to case studies of three human diseases, and the results show that our method performs well in inferring potential interaction between miRNAs and diseases. It is expected that QIMCMDA will become an excellent supplement in the field of biomedical research in the future.

## Introduction

MicroRNAs (miRNAs) are a type of single-stranded small non-coding RNA (∼22 nt) that play an important role in gene regression by interfering with post-transcriptional regulation (Filipowicz et al., 2008; Bartel, 2009). Lee et al. (1993) discovered the first miRNA lin-4 in *Caenorhabditis elegans*, and since then, 1000s of currently annotated miRNAs have been found in various species from plants, animals to viruses (Jopling et al., 2005; Kozomara and Griffiths-Jones, 2011). More and more evidence have shown that miRNA is an important component in cells and may play an important role in a variety of biological processes including cell growth (Ambros, 2003), immune response (Taganov et al., 2006), cell proliferation and differentiation (Chen et al., 2004, 2006), cell development, cell cycle regulation (Carleton et al., 2007), inflammation (Urbich et al., 2008), apoptosis (Petrocca et al., 2008), and stress response (Leung and Sharp, 2010). Many studies have shown that miRNA abnormalities are associated with various human diseases, such as cancer, Alzheimer’s disease, and diabetes (Iorio et al., 2005; Nunez-Iglesias et al., 2010; Catto et al., 2011; Guay et al., 2011; Farazi et al., 2013). For example, there is evidence that MicroRNA-155 regulates colon cancer cell proliferation, cell cycle, apoptosis, migration, and targets CBL (Yu et al., 2017). miR-21 negatively regulates Pdcd4 and inhibits TPA-induced tumor transformation (Asangani et al., 2008). MicroRNA-494 has become a major epigenetic regulator in aggressive human hepatocellular carcinoma neoplasms (Chuang et al., 2005). miR-146a is a tumor suppressor that inhibits NF-κB activity related to the promotion and inhibition of tumor growth (Li et al., 2014b). This makes miRNAs increasingly recognized as key regulators in gene expression (Niu et al., 2019). Finding the association of miRNA-disease is an important field of biomedicine. It not only helps humans understand the mechanism of diseases, but also helps the discovery, prognosis, diagnosis, treatment, and prevention of human complex diseases (Calin and Croce, 2006; Tricoli and Jacobson, 2007; Cho, 2010; Jiang et al., 2010).

However, the identification of miRNA-disease associations using traditional biological methods is often costly (Chen et al., 2018). Therefore, the use of mathematical and computational tools to predict potential miRNA-disease associations based on various experimentally validated association datasets is a hot issue. Through the integration and collection of data from a large number of biological experiments, there are now multiple databases related to miRNA-disease relationships such as HMDD and dbDEMC (Lu et al., 2008; Yang et al., 2010; Li et al., 2014a). In recent years, a large number of miRNA-disease association prediction methods have been proposed. For instance, Chen and Yan (2014) proposed a regularized least squares model (RLSMDA) to predict miRNA-disease associations. This model is a semi-supervised model that learns in the miRNA space and disease space respectively, and then combines to get the final prediction score. However, it should be pointed out that the parameter selection of this model is more difficult, and the combined form of the two spatial scores can be improved in the end. Xu et al. (2011) proposed a method based on support vector machine (SVM) to predict the interaction between miRNA and the disease. However, the current database rarely provides data for non-cancer miRNAs. Therefore, the main problem of the model is the lack of negative samples, which will make the supervised learning model unsuitable for the prediction of large-scale disease-miRNA interactions. Obtaining large numbers of negatively associated samples is still difficult (Guan et al., 2020). Chen et al. (2012) adopted restart random walk (RWRMDA) to predict the potential miRNA-disease interaction, which restarted the known miRNA-disease interaction network, using random walks on miRNA functional similarity network to predict potential miRNA-disease interaction. However, this method is not applicable to the prediction of new diseases that are not related to any miRNA. Chen (2018) introduced the induction matrix completion model (IMCMDA) for the prediction of miRNA disease association based on the known miRNA-disease association matrix, miRNA functional similarity and disease semantic similarity matrix. However, this method is too sensitive to the noise in the data, which affects its performance. Chen et al. (2016b) introduced the model of Within and Between Score for MiRNA-Disease Association prediction (WBSMDA) by a combination of integrated similarity and known miRNA-disease associations. Chen et al. (2018) introduced the MiRNA-disease association prediction (TLHNMDA) model based on three-layer heterogeneous network inference, which integrates multi-level data about miRNA, disease, lncRNA and their associated information into three layers heterogeneous network to determine the relationship between miRNA and disease Potential biological connection. Zhao et al. (2018) proposed a novel computational model of Symmetric Non-negative Matrix Factorization for MiRNA-Disease Association prediction (SNMFMDA) to reveal the relation of miRNA-disease pairs. Compared to the direct use of the integrated similarity in previous computational models, the integrated similarity needs to be interpolated by symmetric non-negative matrix factorization (SymNMF) before application in SNMFMDA. Jihwan Ha et al. (2020) present IMIPMF, a novel method for predicting miRNA–disease associations using probabilistic matrix factorization (PMF), which is a machine learning technique that is widely used in recommender systems. Zhu et al. (2020) proposed a new computational model based on biased heat conduction for MiRNA-Disease Association prediction (BHCMDA),which can achieve the AUC of 0.8890 in LOOCV.

We hope to use a simple and effective method for prediction. Here, we proposed a new matrix completion algorithm based on the q-kernel function to predict new miRNA disease associations (QIMCMDA). This model used miRNA q-kernel similarity, disease q-kernel similarity, known miRNA disease associations, and miRNA functional similarity. A matrix decomposition algorithm based on KL divergence was used to complement missing miRNA-disease associations. Here we used the receiver operating characteristic (ROC) curve as an evaluation index to evaluate the effectiveness of QIMCMDA. For known miRNA-disease associations downloaded from HMDD V2.0, the relevant data was cross-validated using the method of leave-one-out cross-validation (LOOCV) and fivefold cross-validation, and compared with the four previous classic methods (TLHNMDA, WBSMDA, RLSMDA, and IMCMDA). In addition, case studies were conducted on three common human diseases (Breast Neoplasms, Carcinoma Hepatocellular, Colon Neoplasms). All candidate miRNAs for these three diseases were ranked according to the predicted scores of QIMCMDA. Then the top 50 predicted miRNAs of these three diseases were verified in dbDEMC and HMDD 3.2 respectively. As a result, 46, 45, and 48 of the top 50 potentially relevant miRNAs for the three diseases were confirmed. These results indicated the effectiveness of QIMCMDA in predicting potential miRNA-disease associations.

## Materials and Methods

### Human MiRNA-Disease Associations

In this study, we used human disease-miRNA associations in the HMDD v2.0 database, the dataset contains 383 diseases, 495 miRNAs, and 5430 high-quality experimentally verified human miRNA-diseases associations (Chen et al., 2018). We defined the adjacency matrix *A* ∈ *R*^*nd*^∗^*nm*^ as follows:

$$A\,\left(d\,\left(i\right),m\,\left(j\right)\right)=\left\{ \begin{array}{c} 1\;d\,i\,s\,e\,a\,s\,e\,d\,\left(i\right)\,h\,a\,s\,a\,s\,s\,o\,c\,i\,a\,t\,i\,o\,n\,w\,i\,t\,h\,m\,i\,R\,N\,A\,m\,\left(j\right)\\ 0\;d\,i\,s\,e\,a\,s\,e\,d\,\left(i\right)\,h\,a\,s\,n\,o\,a\,s\,s\,o\,c\,i\,a\,t\,i\,o\,n\,w\,i\,t\,h\,m\,i\,R\,N\,A\,m\,\left(j\right) \end{array}\right.\,\left(1\right)$$

### MiRNA Functional Similarity

MiRNA functional similarity score was calculated by Wang et al. (2010) based on the hypothesis that similarly functional miRNAs tend to be associated with diseases with similar phenotypes. Thanks to their work, we obtained from http://www.cuilab.cn/files/images/cuilab/misim.zip downloaded the data. We constructed a matrix *FS*, where the matrix *FS*(*m*(*i*),*m*(*j*)) represents the functional similarity between miRNAs*m*(*i*)and *m*(*j*).

### Disease Semantic Similarity

#### Disease Semantic Similarity 1

A Directed Acyclic Graph (*DAG*) was constructed to describe a disease based on the MeSH descriptors downloaded from the National Library of Medicine (Lipscomb, 2000). The *DAG* of disease *D* included not only the ancestor nodes of *D* and *D* itself but also the direct edges from parent nodes to child nodes. The semantic score of disease *D* could be defined by the following equation:

$$D\,V\,1\,\left(D\right)=\sum_{d\in T\,\left(D\right)}D\,1_{D}\,\left(d\right)\;\left(2\right)$$

we defined the contribution score of disease *d* in *DAG(D)* to the disease *D* by:

$$\left\{ \begin{array}{c} D\,1_{D}\,\left(d\right)=1\;i\,f\,\,d=D\\ D\,1_{D}\,\left(d\right)=m\,a\,x\,\left\{{△}^{*}\,D\,1_{D}\,\left(d^{\prime }\right)|d^{\prime }\in c\,h\,i\,l\,d\,r\,e\,n\,o\,f\,d\right\}\;i\,f\,\,d\neq D \end{array}\right.\;\left(3\right)$$

Δ is the semantic contribution factor. The contribution score of disease is decreased as the distance between *D* and other diseases increases. Based on the assumption that two diseases with larger shared area of their *DAGs* may have greater similarity score, the semantic similarity score between disease *d*(*i*) and disease *d*(*j*) could be defined by the following equation:

$$S\,S\,1\,\left(d\,\left(i\right),d\,\left(j\right)\right)=\frac{\sum_{t\in T\,\left(d\,\left(i\right)\right)\cap T\,\left(d\,\left(j\right)\right)}\left(D\,1_{\left(d\,\left(i\right)\right)}\,\left(t\right)+D\,1_{d\,\left(j\right)}\,\left(t\right)\right)}{D\,V\,1\,\left(d\,\left(i\right)\right)+D\,V\,1\,\left(d\,\left(j\right)\right)}\;\left(4\right)$$

#### Disease Semantic Similarity 2

From above formula (3), it is easy to see that the diseases in the same layer of *DAG(D)* will make the same contribution to the semantic value of *D*. Moreover, for diseases in the same layer of *DAG(D)*, it is reasonable to assume that the diseases appeared in fewer *DAGs* will be more specific than those diseases appeared in more *DAGs*. Hence, to protrude the contribution of these more specific diseases, the contribution of the node d in *T(D)* to the semantic value of the disease *D* could be obtained according to the following formula as well (Chen, 2018):

$$D\,2_{D}\,\left(d\right)=-\log\left[\frac{\text{the}\,\text{number}\,\text{of}\,\text{DAGs}\,\text{containing}\,\text{d}}{\text{the}\,\text{number}\,\text{of}\,\text{diseases}}\right]\,\left(5\right)$$

Based on the above formula, the semantic value of the disease *D* could be obtained according to the following formula as well:

$$D\,V\,2\,\left(D\right)=\sum_{d\in T\,\left(D\right)}D\,2_{D}\,\left(d\right)\,\left(6\right)$$

Hence, the semantic similarity between two diseases *d*_*i*_ and *d*_*j*_ could be obtained according to the following formula as well:

$$S\,S\,1\,\left(d\,\left(i\right),d\,\left(j\right)\right)=\frac{\sum_{t\in T\,\left(d\,\left(i\right)\right)\cap T\,\left(d\,\left(j\right)\right)}\left(D\,2_{\left(d\,\left(i\right)\right)}\,\left(t\right)+D\,2_{d\,\left(j\right)}\,\left(t\right)\right)}{D\,V\,2\,\left(d\,\left(i\right)\right)+D\,V\,2\,\left(d\,\left(j\right)\right)}\;\left(7\right)$$

### q-Kernel Similarity

Many contributions indicate that the performance of kernel-based learning algorithms largely depends on the choice of kernel (Chapelle et al., 2002; Lanckriet et al., 2002; Nogayama et al., 2003). Boughorbel also proved through experiments that in some applications, kernels with only positive conditions may be better than most classical kernels (Boujemaa et al., 2005). Based on this theory, Zhang et al. (2019) designed a variety of q-Kernel Functions, such as Non-Linear q-Kernel, Gaussian q-Kernel, Laplacian q-Kernel, Rational Quadratic q-Kernel, Multiquadric q-Kernel, Inverse Multiquadric q-Kernel, Wave q-Kernel, and so on. A q-analog is a mathematical expression parameterized by a quantity q that generalizes a known expression and reduces to the known expression. Therefore, after a long period of trial, we have chosen the inverse quadratic square q kernel function as the main method for calculating similarity.

Here we introduce a q-Kernel function (inverse multiquadric q-Kernel) and construct a q-Kernel similarity. Based on the assumption that similar miRNAs are more likely to exhibit interactions with similar diseases and vice versa. The q-Kernel similarity is used to calculate the kernel similarity of miRNA and disease, respectively, based on known miRNA- diseases. The value range of the two parameters c and q of the function is between 0 and 1.

$$H_{q}\,\left(x,y\right)=\frac{1}{1-q}\,\left(q^{-\frac{1}{c}}-q^{-\frac{1}{\sqrt{{||x-y||}^{2}+c^{2}}}}\right)\;\left(8\right)$$

### Similarity Calculation of miRNA Based on q-Kernel

In previous work, we obtained a similarity network between two miRNAs. But the integrity of this network is only 0.2058, and too many missing values make it impossible for us to use this network directly. Here, the q-kernel function is used to complete the matrix. First, the obtained q-kernel distance needs to be normalized and scaled to [0,1], because the similarity network value of the previous miRNA is between [0,1]. Then we used the 1-*H*_*q*_ to convert the kernel distance into the similarity and a q-kernel similarity network of miRNA is obtained, which is called QM. The similarity of MiRNA is constructed as follows:

$$S_{m}\left(m\left(i\right),m\left(j\right)\right)\{\begin{array}{cc} \begin{array}{ccc} \omega FS\left(m\left(i\right),m\left(j\right)\right)+(1-\omega )QM(m(i),\;m(j)) & & m\left(i\right)\;and\;m\left(j\right)\\ & & has\;similarity\\ QM\left(m\left(i\right),m\left(j\right)\right) & & otherwise \end{array} & \left(9\right) \end{array}$$

The *ω* is a weighting parameter defined as limiting the effect of FS and QM on miRNA similarity. Set *ω* to 0.01 through training. The greater similarity between miRNAs, the more similar the miRNAs are.

### Network Similarity Calculation for Diseases Based on q-Kernel

We used the same method as the miRNA similarity network to build the disease similarity network QD. Then integrated QD with disease semantic similarities *SS1* and *SS2*:

$$S_{d}\left(d\left(i\right),d\left(j\right)\right)=\{\begin{array}{cc} \begin{array}{ccc} \omega SS(d(i),d(j))+(1-\omega )QD(d(i),d(j)) & & d(i)\;and\;d(j)\\ & & has\;similarity\\ QD(d\left(i\right),d(j)) & & otherwise \end{array} & \;(10) \end{array}$$

$$S\,S\,\left(d\,\left(i\right),d\,\left(j\right)\right)=\frac{S\,S\,1\,\left(d\,\left(i\right),d\,\left(j\right)\right)+S\,S\,2\,\left(d\,\left(i\right),d\,\left(j\right)\right)}{2}\;\left(11\right)$$

We set the parameter values of c and q through training, that is, c = 0.1 and q = 0.6. Finally, we obtained two kernel similarity matrices, *S*_*m*_ and *S*_*d*_.

### Matrix Completion

After integrated various known data and similarity calculations of q-kernel, we can obtain human miRNA-disease correlation matrix A (Matrix density is 0.028), disease similarity matrix*S*_*d*_, miRNA similarity matrix *S*_*m*_. Our goal is to deduce undiscovered miRNA-disease associations based on this known information. Here we use *S*_*d*_ ∈ *R*^*nd*^∗^*nd*^ as the feature matrix of *nd* diseases, and *S*_*m*_ ∈ *R*^*nm*^∗^*nm*^ as the feature matrix for miRNAs. *S*_*d*_(*i*)denote the feature vector of disease *d*(*i*), and *S*_*m*_(*j*) denote the feature vector of miRNA *m*(*j*). The main idea of QIMCMDA is to complement the two feature matrices *S*_*d*_ and *S*_*m*_ by the similarity of the q-kernel, and then supplement the missing elements under the restriction of the association matrix *A* to obtain the potential associations. Finally, the recovery matrix *Z* is obtained, and the form of *Z* is *Z* = *S*_*d*_*WH*^*T*^*S*_*m*_. where *W* ∈ *R*^*nd*^∗^*r*^ and*H* ∈ *R*^*r*^∗^*nm*^, *r* is the desired rank which is equal to*min*⁡(*rank*(*W*),*rank*(*H*)). The parameter *r* mainly affects the convergence speed of the algorithm, and has little effect on the results. The matrices *W* and *H* can be obtained as a solution to the following optimization problems.

$$\min_{W\cdot H}\emptyset =\;\sum {}_{i=1}^{nd}\sum {}_{j=1}^{nm}(A_{ij}\ln\frac{A_{ij}}{S_{d}*W*H*S_{m}}-A_{ij}+{\left(S_{d}*W*H*S_{m}\right)}_{ij})$$

s.t.W≥0,H≥0           (13)

*W* and *H* were set to random dense matrices, and then the alternating gradient descent method is used to update iterations *W* and *H*.

$$W\leftarrow \frac{W^{*}\,\left[\left(\frac{S_{d}*A}{S_{d}*W*H*S_{m}}\right)*S_{m}*H^{\prime }\right]}{S_{d}*ONES*S_{m}*H{}^{\prime }}\;\left(14\right)$$

$$H\leftarrow \frac{H^{*}\,\left[W^{\prime }*S_{d}*\left(\frac{A^{*}\,S_{m}}{S_{d}*W*H*S_{m}}\right)\right]}{W^{\prime }*S_{d}*O\,N\,E\,S*S_{m}}\;\left(15\right)$$

Through the alternating gradient descent algorithm, *W* and *H* will stabilize and stop the iteration after reaching the maximum number of iterations. Here, the maximum number of iterations is set to 100. *ONES* is a matrix, all its elements are 1. It is used to multiply two matrixes of different ranks. We can use *W* and *H* to calculate the predicted score between disease *d*(*i*) and miRNA *m*(*j*) by the following formula(Symbol meaning can refer to Table 1).

**TABLE 1:** Notations.

**Symbol**	**Description**
*nm*	number of miRNAs
*nd*	number of diseases
*A* ∈ *R*^*nd*^∗^*nm*^	miRNA-diseases associations matrix
*S*_*m*_ ∈ *R*^*nm*^∗^*nm*^	miRNA similarity matrix
*S*_*d*_ ∈ *R*^*nd*^∗^*nd*^	disease similarity matrix
*W* ∈ *R*^*nd*^∗^*r*^	alternating iteration matrix in matrix factorization
*H* ∈ *R*^*r*^∗^*nm*^	alternating iteration matrix in matrix factorization

$$S\,c\,o\,r\,e\,\left(d\,\left(i\right),m\,\left(j\right)\right)=S_{d}\,\left(i\right)\,W\,H\,S_{m}\,\left(j\right)\;\left(16\right)$$

**FIGURE 1:**
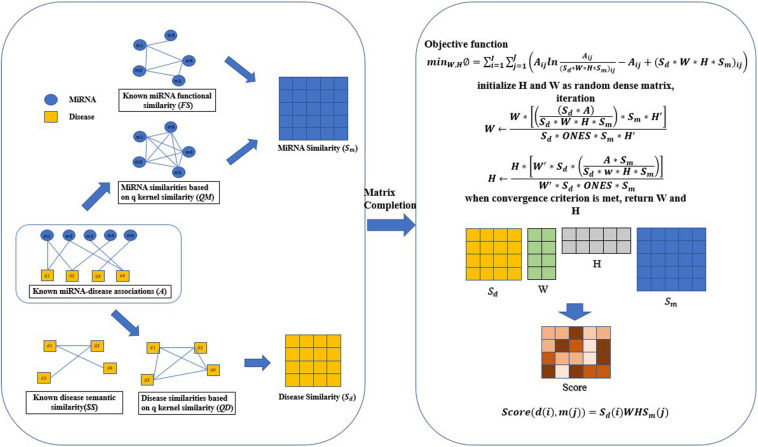
Flowchart of QIMCMDA model to infer the potential miRNA-disease associations. ONES is an all-ones matrix of rank nd^∗^nm.

The specific implementation process of QIMCMDA is shown in Figure 1.

## Results

**FIGURE 2:**
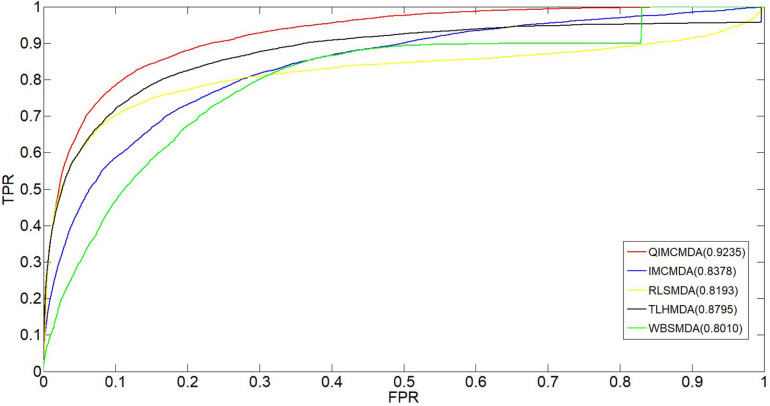
Performance comparison between QIMCMDA and other benchmark methods (RLSMDA, IMCMDA, TLHNMDA, WBSMDA) on AUC of LOOCV.

**FIGURE 3:**
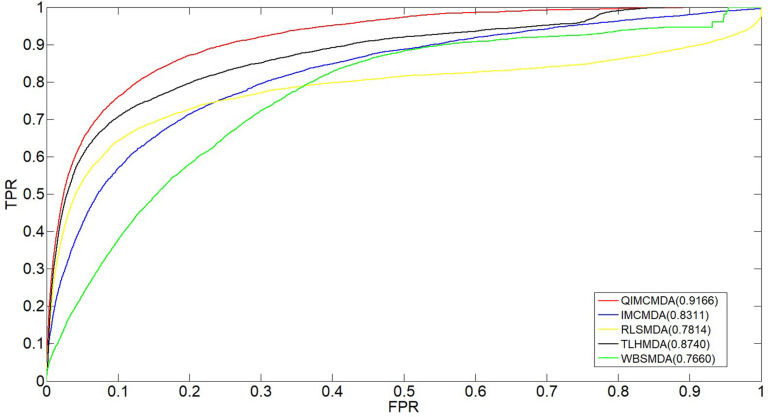
Performance comparison between QIMCMDA and other benchmark methods (RLSMDA, IMCMDA, TLHNMDA, WBSMDA) on AUC of fiveflod CV.

We used 5,430 miRNA-disease associations from HMDD v2.0 as the gold standard dataset, and we used LOOCV and fivefold CV to test the effectiveness of QIMCMDA. In addition, QIMCMDA will be compared with four other methods IMCMDA (Chen, 2018), RLSMDA (Chen and Yan, 2014), TLHNMDA (Chen et al., 2018), WBSMDA (Chen et al., 2016b) to evaluate the predictive ability of QIMCMDA (see Table 2). In the framework of the LOOCV evaluation, 5430 miRNA-disease associations in the data set are considered as test samples one by one, the other remaining samples are considered as training samples, and samples with unknown associations are considered as candidate samples. Through the calculation of the model, we can obtain the prediction score, and then rank and record according to the prediction score. The process of fivefold CV is similar to LOOCV. The miRNA-disease association of the golden data set was randomly divided into five groups, one of which was selected as the test set in turn, and the rest as the training set. Candidate sample settings are the same as LOOCV. Then rank and record the predicted scores for each test sample. Figure 2 shows a comparison of the prediction performance based on the overall AUC value of LOOCV. As a result of LOOCV, the AUC of QIMCMDA is 0.9235, and the AUC values obtained by IMCMDA, RLSMDA, TLHNMDA and WBSMDA are 0.8378, 0.8193, 0.8795, 0.8010, respectively. For fivefold QIMCMDA, IMCMDA, RLSMDA, TLHNMDA and WBSMDA 10 times were performed, and the average AUC and standard deviation were recorded as 0.9170 ± 0.0006, 0.8311 ± 0.0006, 0.7814 ± 0.0020, 0.8735 ± 0.0010,0.7980 ± 0.0009, respectively (see Figure 3).

**TABLE 2:** Under the fivefold CV and LOOCV verification framework, the performance of QIMCMDA and other benchmark methods.

**Methods**	**LOOCV**	**Fivefold CV**
QIMCMDA	0.9235	0.9170 ± 0.0006
IMCMDA	0.8378	0.8311 ± 0.0006
RLSMDA	0.8193	0.7814 ± 0.0020
TLHNMDA	0.8795	0.8735 ± 0.0010
WBSMDA	0.8010	0.7980 ± 0.0009

### Parameter Analysis

There are several hyper-parameters in QIMCMDA that need to be tuned, i.e., c, q, w, k. We use a random search strategy to select hyper-parameters from fixed ranges (Zhang et al., 2020). c and q are parameters for adjusting the q-Kernel function. In this study, the value of c is selected from {0.1,0.2,0.3,0.4,0.5,0.6,0.7,0.8,0.9,1}, and the value of q is selected from {0.1,0.2,0.3,0.4,0.5,0.6,0.7,0.8,0.9}. q can’t be equal to 1. ω is the weight parameter used to integrate similarity. Here, ω is selected from {0.01,0.05,0.1,0.15,0.2,0.3,0.4,0.5,0.8,1}. Next, we show the influence of the these parameters under the fivefold CV.

**FIGURE 4:**
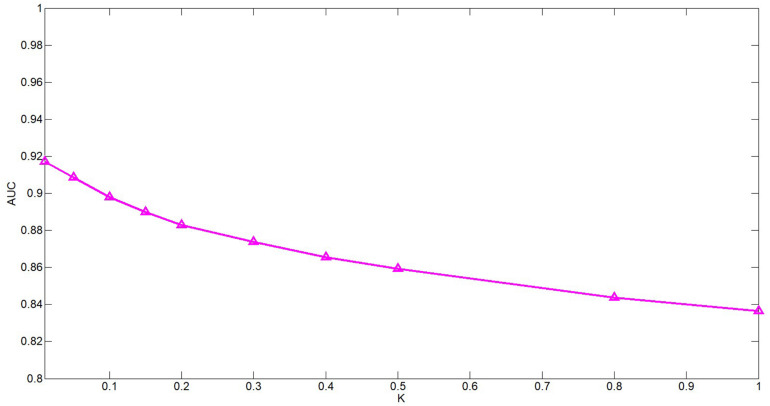
Performance of QIMCMDA with different values of ω under fivefold CV.

**FIGURE 5:**
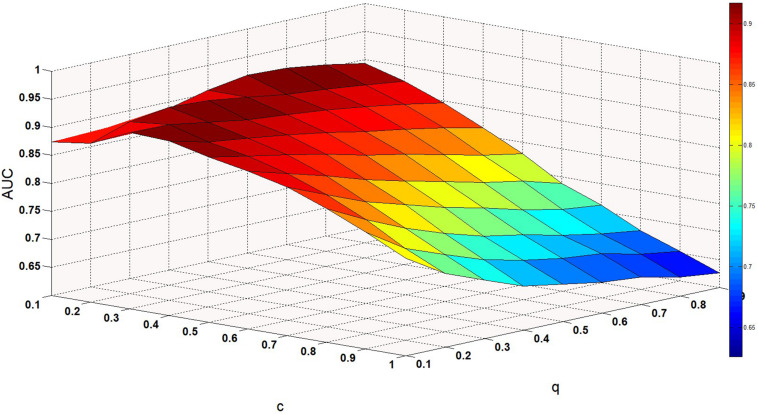
Performance of QIMCMDA with different values of c and q under fivefold CV.

The k is a potential feature size. In our test, the impact of this variable is actually very small, but we still decided to use PCA to calculate the cumulative contribution rate to obtain the most appropriate k value. This method is in the paper by Wang et al. (2017). It has been well-verified. In this article, the cumulative contribution rate of 95% is used to select the PC, and the final k is set 114.

ω is a weight parameter used to integrate the similarity matrix. Figure 4 shows the effect of changes in ω on AUC when other parameters are fixed. When ω = 0.01, AUC takes the maximum value. When c = 0.1, q = 0.6, the model can achieve the best effect (see Figure 5).

### Case Study

In this article, we used case studies to further demonstrate the effectiveness of QIMCMDA. We performed case studies on three diseases: Breast Neoplasms, Carcinoma Hepatocellular, and Colon Neoplasms. These diseases were selected in our case study because they all have high incidence and insignificant early symptoms. In addition, they have been considered as case studies in many previous publications (Guan et al., 2020). Our case study used HMDD v2.0 as the training database for QIMCMDA. HMDD 3.2 and dbDEMC (Lu et al., 2008; Yang et al., 2010; Li et al., 2014a) serve as validation databases to confirm the predicted potential associations. Compared with the previous 2.0 version, the 3.2 version contains more than double the association between human diseases and miRNAs, the classification of evidence is more clear, and there is a clear third-party annotation for each association. The differentially expressed miRNA database (dbDEMC) in human cancer is a comprehensive database microRNA (miRNA) designed to store and display differentially expressed human cancers detected by high-throughput methods. The database collected a total of 209 newly released data sets from Gene Expression Omnibus (GEO) and The Cancer Genome Atlas (TCGA). The current version contains data from 436 biological experiments, including 2224 differentially expressed miRNAs in 36 cancer types. We only perform ranking verification on candidate miRNAs of interest, so training samples are not in the final result. In other words, the miRNA disease associations obtained from the predicted list do not overlap with the known 5430 associations.

Breast Neoplasms is one of the most common malignancies in women. With more than 2 million new cases worldwide each year, it ranks second among the world’s major cancer types (Jemal et al., 2017). More than half of these cases occurred in industrialized countries (Parkin et al., 2005). It was one of the leading causes of death among women aged 20–59 (Siegel et al., 2015). With the development of biological technology, researchers have found more miRNAs related to Breast Neoplasms. Our results are supported by third-party annotations in two databases, HMDD3.2 and dbDEMC. For example, miR-150 and miR-372 can promote the proliferation and growth of Breast Neoplasms cells by targeting the pro-apoptotic purinergic P2X7 receptor and LATS2 respectively (Huang et al., 2017; Cheng et al., 2018). MicroRNA-130a targets RAB5A to inhibit the proliferation, invasion and migration of Breast Neoplasms cells (Pan et al., 2015). miR-494 targets CXCR4 through the Wnt/β-catenin signaling pathway, thereby inhibiting Breast Neoplasms progression in vitro (Song et al., 2015). The increased miR-451 expression may negatively regulate Bcl-2 mRNA and protein expression, which in turn affects caspase 3 protein expression and accelerates Breast Neoplasms cell apoptosis (Gu et al., 2015). MiR-449a inhibits cell migration and invasion in Breast Neoplasms by targeting PLAGL2 (Wang et al., 2018). We selected the top 50 in the results and verified them with two databases, HMDD 3.2 and dbDEMC. It was found that 10 of the first 10 predictions and 46 miRNAs of the first 50 predictions were verified (see Table 3).

**TABLE 3:** Prediction results of the top 50 predicted Breast Neoplasms-related miRNAs based on known associations in HMDD V2.0.

**miRNA**	**Evidence**	**miRNA**	**Evidence**
hsa-mir-151	HMDD3.2	hsa-mir-663	dbDEMC
hsa-mir-30e	HMDD3.2	hsa-mir-382	dbDEMC
hsa-mir-92b	HMDD3.2	hsa-mir-494	HMDD3.2
hsa-mir-451	HMDD3.2	hsa-mir-575	HMDD3.2
hsa-mir-130a	HMDD3.2	hsa-mir-658	dbDEMC
hsa-mir-192	HMDD3.2	hsa-mir-181d	dbDEMC
hsa-mir-98	HMDD3.2	hsa-mir-376a	HMDD3.2
hsa-mir-372	HMDD3.2	hsa-mir-211	dbDEMC
hsa-mir-32	HMDD3.2	hsa-mir-484	HMDD3.2
hsa-mir-106a	HMDD3.2	hsa-mir-455	Unconfirmed
hsa-mir-130b	HMDD3.2	hsa-mir-432	dbDEMC
hsa-mir-99b	dbDEMC	hsa-mir-381	HMDD3.2
hsa-mir-95	dbDEMC	hsa-mir-99a	HMDD3.2
hsa-mir-28	dbDEMC	hsa-mir-154	dbDEMC
hsa-mir-150	HMDD3.2	hsa-mir-523	dbDEMC
hsa-mir-186	dbDEMC	hsa-mir-526b	HMDD3.2
hsa-mir-15b	HMDD3.2	hsa-mir-507	Unconfirmed
hsa-mir-142	HMDD3.2	hsa-mir-525	Unconfirmed
hsa-mir-449b	dbDEMC	hsa-mir-660	HMDD3.2
hsa-mir-198	dbDEMC	hsa-mir-181c	HMDD3.2
hsa-mir-196b	HMDD3.2	hsa-mir-300	dbDEMC
hsa-mir-491	HMDD3.2	hsa-mir-297	dbDEMC
hsa-mir-449a	HMDD3.2	hsa-mir-136	dbDEMC
hsa-mir-424	HMDD3.2	hsa-mir-331	HMDD3.2
hsa-mir-212	HMDD3.2	hsa-mir-512	Unconfirmed

Hepatocellular carcinoma (HCC), one of the most common malignancies worldwide (Yegin et al., 2016), was also the main cause of cancer in men under 60 in China (Chen et al., 2016a). MiRNAs have important roles in the treatment of HCC and have been corroborated. For example, related in vitro experiments have further confirmed the anti-tumor effect of miR-132 in HCC (Liu et al., 2015; Zhang et al., 2016). The newly identified miR-429-CRKL axis represents a new potential therapeutic target for HCC therapy (Guo et al., 2018). MicroRNA-23b inhibits epithelial–mesenchymal transition (EMT) and metastasis of Hepatocellular Carcinoma by targeting Pyk2 (Cao et al., 2017). MicroRNA-494 is a major epigenetic regulator of microRNAs for multiple invasion inhibitors by targeting 10 11 translocation 1 in aggressive human Hepatocellular Carcinoma (Chuang et al., 2005). MicroRNA-340 inhibits the proliferation and invasion of Hepatocellular Carcinoma cells by targeting JAK1 (Yuan et al., 2017). Therefore, 10 of the top 10 predicted miRNAs and 45 of the top 50 predicted miRNAs were confirmed by experimental literature from the dbDEMC and HMDD3.2 (see Table 4).

**TABLE 4:** Prediction results of the top 50 predicted Carcinoma Hepatocellular-related miRNAs based on known associations in HMDD V2.0.

**miRNA**	**Evidence**	**miRNA**	**Evidence**
hsa-mir-132	HMDD3.2	hsa-mir-516a	unconfirmed
hsa-mir-429	HMDD3.2	hsa-mir-663	dbDEMC
hsa-mir-34b	HMDD3.2	hsa-mir-340	HMDD3.2
hsa-mir-151	HMDD3.2	hsa-mir-28	dbDEMC
hsa-mir-30e	HMDD3.2	hsa-mir-186	HMDD3.2
hsa-mir-367	HMDD3.2	hsa-mir-575	HMDD3.2
hsa-mir-339	dbDEMC	hsa-mir-658	dbDEMC
hsa-mir-9	HMDD3.2	hsa-mir-452	HMDD3.2
hsa-mir-215	HMDD3.2	hsa-mir-193b	HMDD3.2
hsa-mir-451	HMDD3.2	hsa-mir-196b	dbDEMC
hsa-mir-194	HMDD3.2	hsa-mir-494	HMDD3.2
hsa-mir-302a	dbDEMC	hsa-mir-449a	HMDD3.2
hsa-mir-32	HMDD3.2	hsa-mir-424	HMDD3.2
hsa-mir-204	HMDD3.2	hsa-mir-520c	HMDD3.2
hsa-mir-135b	HMDD3.2	hsa-mir-382	unconfirmed
hsa-mir-95	HMDD3.2	hsa-mir-301b	dbDEMC
hsa-mir-488	dbDEMC	hsa-mir-510	unconfirmed
hsa-mir-302d	HMDD3.2	hsa-mir-376c	unconfirmed
hsa-mir-23b	HMDD3.2	hsa-mir-455	HMDD3.2
hsa-mir-133a	HMDD3.2	hsa-mir-206	HMDD3.2
hsa-mir-299	HMDD3.2	hsa-mir-137	HMDD3.2
hsa-mir-143	HMDD3.2	hsa-mir-211	HMDD3.2
hsa-mir-153	HMDD3.2	hsa-mir-154	HMDD3.2
hsa-mir-516b	Unconfirmed	hsa-mir-27b	HMDD3.2
hsa-mir-383	dbDEMC	hsa-mir-523	dbDEMC

Colon Neoplasms are the most common type of gastrointestinal cancer (Jemal et al., 2011; Ogata-Kawata et al., 2014). Siegel et al. (2018), there were 97,220 new cases in the United States alone, and approximately 50,630 patients died. A variety of miRNAs have been experimentally confirmed to be associated with colon neoplasms. For example, MicroRNA-155 regulates Colon Neoplasms cell proliferation, cell cycle, apoptosis, migration and targets CBL (Yu et al., 2017). MicroRNA-21 induces stem cells by down-regulating transforming growth factor beta receptor 2 (TGFbetaR2) in Colon Neoplasms cells (Yu et al., 2012). Let-7 is also involved in the development of Colon Neoplasms (Williams, 2008). MicroRNA-221 promotes Colon Neoplasms cell proliferation in vitro (Sun et al., 2011). MicroRNA-34a inhibits the migration and invasion of Colon Neoplasms cells by targeting Fra-1 (Wu et al., 2012). Verification of dbDEMC and HMDD3.2 confirmed 10 of the first 10 predictions and 48 miRNAs of the first 50 predictions (see Table 5).

**TABLE 5:** Prediction results of the top 50 predicted Colon Neoplasms-related miRNAs based on known associations in HMDD V2.0.

**miRNA**	**Evidence**	**miRNA**	**Evidence**
hsa-mir-143	HMDD3.2	hsa-mir-200b	HMDD3.2
hsa-mir-106b	HMDD3.2	hsa-mir-24	HMDD3.2
hsa-mir-21	HMDD3.2	hsa-mir-1	HMDD3.2
hsa-mir-128	HMDD3.2	hsa-mir-205	HMDD3.2
hsa-mir-18a	HMDD3.2	hsa-mir-29b	HMDD3.2
hsa-mir-9	dbDEMC	hsa-let-7b	HMDD3.2
hsa-mir-155	HMDD3.2	hsa-mir-31	HMDD3.2
hsa-mir-181a	HMDD3.2	hsa-mir-223	HMDD3.2
hsa-mir-494	unconfirmed	hsa-let-7c	HMDD3.2
hsa-mir-483	HMDD3.2	hsa-mir-15a	HMDD3.2
hsa-let-7a	HMDD3.2	hsa-mir-200c	HMDD3.2
hsa-mir-125b	HMDD3.2	hsa-mir-222	HMDD3.2
hsa-mir-146a	HMDD3.2	hsa-mir-199a	HMDD3.2
hsa-mir-34a	HMDD3.2	hsa-mir-30b	HMDD3.2
hsa-mir-210	HMDD3.2	hsa-mir-141	HMDD3.2
hsa-mir-16	HMDD3.2	hsa-mir-200a	HMDD3.2
hsa-mir-146b	dbDEMC	hsa-let-7e	HMDD3.2
hsa-mir-221	HMDD3.2	hsa-mir-196a	HMDD3.2
hsa-mir-93	HMDD3.2	hsa-mir-142	HMDD3.2
hsa-mir-92a	HMDD3.2	hsa-let-7f	HMDD3.2
hsa-mir-20b	dbDEMC	hsa-mir-34c	Unconfirmed
hsa-mir-19a	HMDD3.2	hsa-let-7i	HMDD3.2
hsa-mir-29a	HMDD3.2	hsa-let-7d	HMDD3.2
hsa-mir-18b	HMDD3.2	hsa-let-7g	HMDD3.2

## Discussion

Research on the potential prediction of miRNA-disease associations will help us to understand the pathogenesis and treatment of the disease more deeply. Especially for cancer, targeted therapy by regulating miRNA may be a breakthrough point for future treatment. In this paper, we developed an algorithm for miRNA-disease association prediction (QIMCMDA), which mainly introduced the q-kernel function to complete the similarity information required. The QIMCMDA model is based on the known miRNA disease association and miRNA functional similarity network. First, calculated and completed the miRNA similarity network and the disease similarity network using the q-kernel function. Then used the matrix decomposition method to calculate the prediction score for each sample, and finally sort the scores. The AUC of QIMCMDA based on LOOCV is 0.9235, showing better performance than previous methods. In addition, experimental literature has confirmed the validity of potential miRNA-disease association predictions for three major human diseases: Breast Neoplasms, Carcinoma Hepatocellular, Colon Neoplasms).

The reasons for the reliable performance of QIMCMDA are as follows: the key advantage of QIMCMDA is that it utilizes the functional similarity of known miRNAs in combination with q-kernel similarity as features of diseases and miRNAs to complete the association of missing miRNAs and diseases. And the use of alternating gradient descent algorithm to search for the optimal solution can ensure the reliability of disease feature vectors and miRNA feature vectors. In addition, the overall complexity of our method from the construction of the network to the final prediction score calculation is low, and the operation is simple and easy to reproduce. QIMCMDA has a short running time and is suitable for large-scale data research. It is a simple and effective method. Finally, QIMCMDA is a semi-supervised model that does not require negative samples, reducing the difficulty of model construction. Compared with methods that require a large number of negative samples, our method has some advantages. However, QIMCMDA currently has some limitations. First of all, there are inevitable noises and outliers in the known materials we use. Second, QIMCMDA used the KL divergence as an error function, which is unstable due to noise and outliers. With the development of the times, database construction will become more and more perfect. As the number of associated data increases, our predictions will become more accurate. In addition, for miRNA or disease without any known associations, our method may be less effective, because the calculation of q-kernel is mainly based on known associations. In the future, we can use a large amount of biological data to further increase the reliability and practicability of the model prediction. And our method can be practiced in other fields such as the interaction between microorganisms and diseases or the interaction between drugs and targets.

## Data Availability Statement

All datasets generated for this study are included in the article/supplementary material.

## Author Contributions

LW and YZ conceived the study. LW, YZ, and YC developed the prediction method and designed the experiments. LW analyzed the result and wrote the manuscript. NZ and WC optimized the flow chart and manuscript structure. All authors reviewed and improved the manuscript.

## Conflict of Interest

The authors declare that the research was conducted in the absence of any commercial or financial relationships that could be construed as a potential conflict of interest.
